# Current status of laparoscopic hepatectomy for the treatment of hepatocellular carcinoma: A systematic literature review

**DOI:** 10.1097/MD.0000000000027826

**Published:** 2021-12-17

**Authors:** Maher Hendi, Jiemin Lv, Xiu-Jun Cai

**Affiliations:** Department of General Surgery, Sir Run Run Shaw Hospital, School of Medicine, Zhejiang University, 3 East Qingchun Road, Hangzhou, Zhejiang Province, China.

**Keywords:** hepatocellular carcinoma, laparoscopic liver resection, review

## Abstract

**Background:**

Laparoscopic hepatectomy (LH) was first introduced in the 1990s and has now become widely accepted for the treatment of hepatocellular carcinoma (HCC). Laparoscopic liver resection (LLR) is considered a safe and effective approach for liver disease. However, the role of laparoscopic hepatectomy in HCC with cirrhosis remains controversial and needs to be further assessed, and the present literature review aimed to review the surgical and oncological outcomes of Laparoscopic hepatectomy (LH). According to Hong and colleagues laparoscopic resection for liver cirrhosis is a very safe and feasible procedure for both ideal cases and select patients with high risk factors [29]. The presence of only 1 of these factors does not represent an absolute contraindication for LH.

**Methods and results:**

We selected 23 studies involving about 1363 HCC patients treated with LH. 364 (27%) patients experienced major resections. The mean operative time was 244.9 minutes, the mean blood loss was 308.1 mL and blood transfusions were required in only 4.9% of patients. There were only 2 (0.21%) postoperative deaths and overall morbidity was 9.9%. Tumor recurrence ranged from 6 to 25 months. The 1-year, 3-year, and 5-year disease free Survival (DFS) rates ranged from 71.9% to 99%, 50.3% to 91.2%, and 19% to 82% respectively. Overall survival rates ranged from 88% to 100%, 73.4% to 94.5%, and 52.6% to 94.5% respectively.

**Conclusions:**

In our summery LH is lower risk and safer than conventional open liver surgery and is just as efficacious. Also, the LH approach decreased blood-loss, operation time, postoperative morbidity and had a lower conversion rate compared to other procedures whether open or robotic. Finally, LH may serve as a promising alternative to open procedures.

## 1 Introduction

Hepatocellular carcinoma (HCC) is the fifth most common cancer worldwide and the sixth most common primary liver malignancy in the United States. HCC is also the second leading cause of cancer related death in the world due to its highly malignant nature.^[1]^ HCC is a common primary malignancy of the liver, characterized by poor prognosis. Discovering tumor-specific prognostic factors to foresee results and improve treatment methods is urgently needed for HCC patients. Hepatic resection remains an essential treatment strategy for HCC patients with early-stage HCC (BCLC stage 0–A) (2). About 80% to 90% of HCC cases arise in a cirrhotic liver.^[2]^ The surgical approach for liver cancer is affected by tumor stage and localization. There is growing evidence that the increasing incidences of HCC in the western world can be attributed to the Hepatitis C Virus (HCV).^[3]^ Surgical resection is the gold standard in the therapeutic management of HCC. However, prognosis remains poor, probably due to late-stage diagnoses. Additional treatment options include radiofrequency ablation, transarterial chemoembolization, and liver transplant.^[4,5]^ The first laparoscopic liver resection (LLR) was reported in 1992, whereas the first LLR for HCC was reported in 1995.^[6,7]^ For many years, the application of laparoscopic hepatectomy (LH) was considered controversial. Progress in laparoscopic techniques and expertise in combination with technologic advances have led to a more widespread adoption of minimally invasive approaches for the resection of HCC over the last 15 years.^[8]^ With advancements in surgical instruments and experience in laparoscopic treatment for benign liver diseases, there has been a growing interest in its application for HCC. When compared with open liver resections (OLR), LLRs are safe with acceptable morbidity and mortality rates for both minor and major liver resections.^[17]^ Improved laparoscopic techniques, better visualization of the operative field using a flexible laparoscope and routine use of a laparoscopic cavitron ultrasonic surgical aspirator to tran-sect the deeper portion of the liver parenchyma have allowed laparoscopic left lateral sectionectomy to be performed more widely.^[10]^

Therefore, this work primarily aims to assess the current indications, advantages and limitations of laparoscopic surgery for HCC resection. We will also discuss the feasibility of LLR and review the global clinical evidence of laparoscopic resection for HCC by assessing reports published before March 2020.

## 2 Materials and methods

### 2.1 Literature search strategy

We conducted a search to identify relevant articles on laparoscopic hepatectomy that have been published in the EMBASE, PubMed, Web of Science, and Cochrane Library databases up to 2019. The search terms included laparoscopic liver resection, laparoscopic hepatectomy, minimally invasive liver procedures, hepatocellular carcinoma there were no restrictions on publication date. Search terms were confined to Title/Abstract: laparoscopic OR “laparoendoscopic.” The reference lists of all selected articles were manually searched to determine if they should be included. All eligible English studies were retrieved. We also reviewed the reference lists of the included studies for further relevant studies. In addition, the ethical approval was not applied in current study because there was no patients privacy or clinical samples.

### 2.2 The standard of laparoscopic liver resection procedures

Over the past decade, LLR has progressed internationally following advances in technology and the increasing experience of liver surgeons. LH has been a well-established treatment option for HCC even in patients with liver cancer. A few years ago, 1 publication described the benefits of the LLR procedure, and more than 9000 procedures were reported in English journals.^[11]^ The selection criteria of laparoscopic liver resection follow the same principles as open surgery. LLR is considered a safe technique with low mortality and morbidity rates, 0% and 15%, respectively.^[12]^

Eventually the LLR technique will be comparable to and as feasible as the open approach. Its techniques have gradually become incorporated into the practices of most liver centers and LLR is now widely accepted for the management of benign and malignant liver tumors.^[13]^

### 2.3 Data extraction and outcome measures

Outcomes included in this review were overall morbidity, major morbidity, over all complications, blood loss, blood transfusion, conversion rate, major/minor resection, operative time, length of hospital stay and 1-, 3-, and 5-year overall survival and Disease Free Survival (DFS) rates. Data on the type of study, number of patients enrolled, patients’ age and sex, tumor size and recurrence rate, and types of surgery were also recorded. Major morbidity was extracted when stated or if the Clavien–Dindo scale was used. Complications were rated as Clavien–Dindo grade I, II, III, IV, or V.

## 3 Results

Twenty three publications that reported on 1363 HCC patients who had underwent LH were selected. A total of 2987 studies were screened out by searching electronic databases.

**Figure 1:**
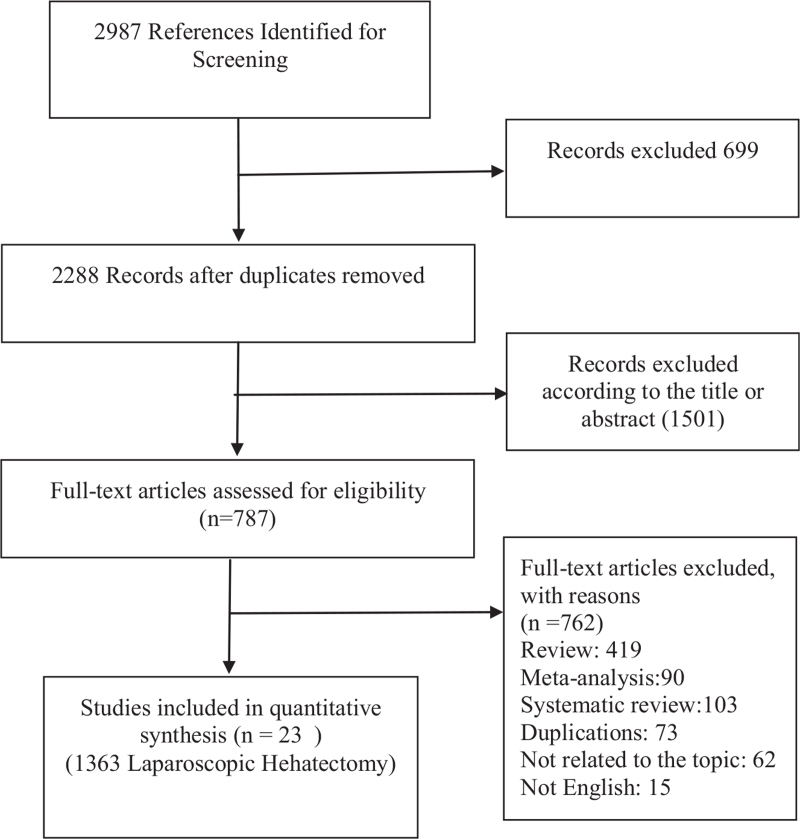
Flow chart illustrating selection process.

Twenty three publications with more than 20 patients per study were selected. Table 1 illustrates the results of the search. Patient characteristics, perioperative results, and oncologic outcomes were analyzed. The detailed steps of our literature search are shown in (Fig. 1).

**Table 1:** Publications on laparoscopic liver resection for hepatocellular carcinoma.^[14–36]^.

Study (year)	No. of patient	Country	Journal	Age	Gender (M/F)	Minor resection	Major resection
Shimada et al, 2001	17	Japan	Surg Endosc	62 ± 9	15/2	17 (100%)	0 (0%)
Laurent et al, 2003	13	France	Arch Surgery	62 ± 9.5	10/3	10 (77%)	3 (23%)
Kaneko et al, 2005	30	Japan	American Journal of Surgery	59	18/12	30 (100%)	0 (0%)
Cherqui et al, 2006	27	France	Annals of Surgery	63	22/5	26 (97%)	1 (0.3%)
Chen et al, 2008	116	Taiwan	Annals of Surgical Oncology	58	92/24	97 (84%)	19 (16%)
Cai et al, 2008	31	China	Surg Endosc	54.2 (23–81)	24/7	20 (65%)	11 (35%)
Sarpel et al, 2009	20	USA	Annals of Surgical Oncology	63 ± 10.3	15/5	20 (100%)	0 (0%)
Yoon et al 2010	69	S. Korea	Surg Endosc	65.6	50/19	60 (87%)	9 (13%)
Truant et al, 2011	36	France	Surg Endosc	66.6 ± 10	31/5	36 (100%)	0 (0%)
Akishige et al, 2013	56	Japan	Surg Endosc	68.5	33/23	45 (80%)	11 (20%)
Memeo et al, 2014	45	France	World Jou.Surgery	62 (34–75)	35/10	30 (66%)	15 (34%)
Ahn et al, 2014	51	S. Korea	Journal of Lap& Adv Sur Tech	58.2–10.4	36/15	49 (96.8%)	2 (3.92%
Komatsu et al, 2016	38	France	Surgical Endoscopy	61.5 (12.2)	34/4	30 (78.9%)	8 (23.6%)
Chen et al, 2017	81	Taiwan	Ann Surgical Oncol	60 (22–89)	57/24	47 (58.1%)	34 (41.9%)
Junhua et al, 2017	126	China	Medicine	51 (21–76)	93/33	60 (47.6%)	66 (52.3%)
Hong et al, 2018	36	China	Surgical Endoscopy	53.5 (26–70	30/6	0 (0%)	36 (100%)
Yusuke et al, 2018	160	Japan	Surgical Endoscopy	69 (39–87)	112/48	NA	NA
Rhu et al, 2018	53	S. Korea	World Jou.Surgery	58.0 ± 8.8	43/10	NA	NA
Yoon. et al, 2019	217	S. Korea	Surgical Endoscopy	56 (±9.65)	170/47	112 (51.6%)	105 (48.39%)
Peng. et al, 2019	33	China	Journal of Lap& Adv Sur Tech	55 (35–76)	28/5	16 (48.5%)	17 (51.5%)
Onoe. et al, 2019	30	Japan	Surgical Endoscopy	70 (50–85)	23/7	5 (16.7%)	25 (83.3%)
Yamamoto et al, 2019	58	Japan	Surgical Endoscopy	71 (34–89)	39/19	NA	NA
Goh. et al, 2019	20	Singapore	World Jou. Surgery	68.5 (67–71)	18/2	18 (90%)	2 (10%)

NA = not available.

**Figure 2:**
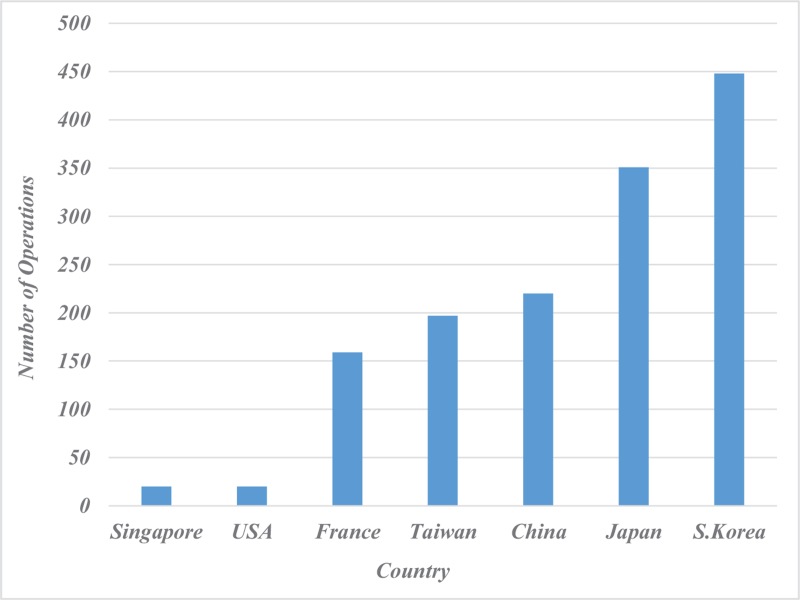
Number of Laparoscopic Hepatectomy by Country.

LH cases were listed by institutions from Japan, France, South Korea, China, Taiwan, Singapore and the USA in order of decreasing frequency (Fig. 2). Table 2 shows short-term and long-term major outcomes for each study. Mortality rates ranged from 0% to 3% with 21 series out of 23 reporting 0% mortality. Considering all the studies, only 2 patients died in the perioperative period and the causes of death were not clear.

**Table 2:** Morbidity and mortality rates in the different studies.^[14–36]^.

Study (year)	Morbidity	Mortality	Clavien I–II	Clavien III–IV–V	Over all complications
Shimada et al, 2001	5.9 (35%)	NA	Not matched	Not matched	5.9 (35%)
Laurent et al, 2003	4 (31%)	0 (0%)	Not matched	Not matched	5 (36%)
Kaneko et al, 2005	NA	NA	NA	NA	10 (33%)
Cherqui et al, 2006	9 (33%)	0 (0%)	NA	NA	2 (7%)
Chen et al, 2008	6 (5%)	0 (0%)	Not matched	Not matched	11.4 (10%)
Cai et al, 2008	0 (0%)	0 (0%)	NA	NA	NA
Sarpel et al, 2009	NA	NA	NA	NA	NA
Yoon et al, 2010	15 (22%)	0 (0%)	NA	NA	21 (30.4%)
Truant et al, 2011	9 (25%)	0 (0%)	NA	NA	6 (16%)
Akishige et al, 2013	23 (41%)	0 (0%)	15 (27%)	8 (14%)	35 (62%)
Memeo et al, 2014	9 (20%)	1 (2%)	4 (10%)	3 (6%)	10 (22.21%)
Ahn et al, 2014	3 (5.88%)	0 (0%)	2 (3.9%)	1 (2%)	3 (5.88%)
Komatsu et al, 2016	Not matched	0 (0%)	7 (18.4%)	5 (13.15%)	12 (31.6%)
Chen et al, 2017	4 (4.9%)	0 (0%)	4 (4.9%)	0 (0%)	4 (4.9%)
Junhua et al, 2017	49 (22.2%)	0 (0%)	10 (7.9%)	2 (1.6%)	28 (22.2%)
Hong et al, 2018	11 (30.6%)	0 (0%)	NA	Not matched	11 (30.6%)
Yusuke et al, 2018	Not matched	1 (3.0%)	Not matched	Not matched	5 (3.12%)
Rhu et al, 2018	Not matched	0 (0%)	4 (7.55%)	1 (20%)	5 (9.4%)
Yoon et al, 2019	14 (6.5%)	0 (0%)	6 (2.8%)	0 (0%)	14 (6.5%)
Peng et al, 2019	4 (12.1%)	0 (0%)	Not matched	Not matched	6 (18.2%)
Onoe et al, 2019	2 (6.7%)	0 (0%)	3 (10%)	2 (6.75%)	2 (6.7%)
Yamamoto et al, 2019	Not matched	0 (0%)	Not matched	Not matched	9 (15.5%)
Goh et al, 2019	2 (10%)	0 (0%)	NA	2 (10%)	NA

NA = not available.

Overall morbidity rates ranged from 4.9% to 41%, with 16 series out of 23 reporting morbidity. Six of the 23 papers did not report morbidity or the number of morbidities was not clear. Classification according to Clavien-Dindo was available for 9 studies and in these series most of the postoperative complications fell within Clavien grade I and II (range: 2.8%–18.4%), while grades III to V were a minority (range: 0%–13.15%). A total of 20 studies reported overall complications and 3 studies reported no overall complications data. The overall complications rate was between 3.12% and 31.6%. (Table 2).

### 3.1 Intra-operative and postoperative outcomes

Intraoperative, Postoperative data are recorded in Table 3 and the mean operative time ranged from 140 to 381 minutes and the median duration of hospitalization ranged from 6 to 10 days. However, the authors Chen, Akishige, and Goh^[18,23,36]^ did not report the length of hospital stays. The median blood loss ranged from 87 to 808.3 mL. The maximum rate of conversion reported was 15%. However, 6 studies have not reported the conversion rates. Blood transfusion was required in 5.22% of patients, only 6 studies did not report blood Transfusions. (Table 3).

**Table 3:** Intra- and postoperative outcomes in different studies.^[14–36]^.

Study (year)	Total operation time (min)	Blood loss (mL)	Conversion (%)	Duration of hospitalization (days)	Blood transfusion (n)
Shimada et al, 2001	325	400	0 (0%)	6.2 ± 4.0	5.9
Laurent et al, 2003	267	620	2 (15%)	15.3 ± 8.6	4
Kaneko et al, 2005]	182	350	1 (3%)	14.9 ± 7.1	NA
Cherqui et al, 2006	240	338	7 (25%)	7.8 ± 2.6	5 (19%)
Chen et al, 2008	156.2	138.9	6 (5%)	NA	8 (7%)
Cai et al, 2008	140.1	502.9	1 (3%)	7.5 ± 5	NA
Sarpel et al, 2009	161	NA	0 (0%)	6 ± 8.5	NA
Yoon et al, 2010	280.9	808.3	5 (7%)	9.9 ± 5.6	23 (33%)
Truant et al 2011	193.4 ± 104	452.2 ± 442	NA	6.5 ± 2.7	1 (2.8%)
Akishige et al, 2013	Not Clear	Not clear	Not clear	Not clear	Not clear
Memeo et al, 2014	140 [45–360]	200 [0–1,500]	Not clear	7 [0–69]	0 (0%)
Ahn et al, 2014	210.7+−131.3	350 (0–432.5)	Not clear	8.2+−4.6	3 (5.9%)
Komatsu et al, 2016	365 (180–600)	100 (20–900)	Not clear	7.5 (3–51)	2 (5.2%)
Chen et al, 2017	343 [140–715]	282 [50–2200]	0 (0%)	7.5 [3–26]	6 (7.4%)
Junhua. et al, 2017	240 (75–590)	200 (20–2500)	0 (0%)	6 (3–21)	6 (4.8%)
Hong et al, 2018	255 (110–500)	300 (50–1000)	0 (0%)	8 (4–22)	1 (3.1%)
Yusuke et al, 2018	217 (43–356)	Not clear	1 (0.8%)	6.5 (3–47)	5 (3.12%)
Rhu et al, 2018	381 (149.0)	Not clear	0 (0%)	8.9 ± 3.6	7 (13.2%)
Yoon. et al, 2019	234.2 (± 102.06)	225.7 (± 362.15)	Not Clear	8.9 (± 2.60)	4 (1.8%)
Peng et al, 2019	225 (80–505)	200 (20–1500)	1 (3.0%)	7 (2–15)	1 (2.9%)
Onoe, et al 2019	276 (125–589)	100 (0–1050)	2 (6.75%)	10 (4–50)	Not Clear
Yamamoto et al, 2019	242 (66–682)	87 (1–798)	0 (0%)	9 (5–45)	Not Clear
Goh et al, 2019	315 (181–395)	200 (100–425)	3 (15%)	Not clear	2 (10%)

MIN = minute, N = number, NA = not available.

### 3.2 Oncologic outcomes and survival

Table 4 shows the outcomes from the selected publications. Tumor size ranged from 1.8 to 4.7 cm. One study did not report tumor size. Resection margin was reported in most of the studies. Only 8 studies reported the average time for the development of tumor recurrence and the range was 2 to 25 months. There was no incidence of metastasis or tumor recurrence. The 1-year, 3-year, and 5-year overall survival rates ranged from 85% to 100%, 73.4 to 94.5%, and 48 to 94.5% respectively.

**Table 4:** Oncologic outcomes and overall survival and disease-free survival in the different studies^[14–36]^.

Study (year)	Study type	Tumour size (cm)	Recurrence (month)	year OS	1-yearDFS	3-year OS	3-year DFS	5-year OS	year DFS
Shimada et al, 2001	PC	2.6 ± 0.9	0	85%	80%	NA	NA	48	40
Laurent et al, 2003	PC	3.35	5 (38%)	89%	46%	89%	46%	NA	NA
Kaneko et al, 2005	PC	3.1	0	97%	87%	NA%	NA%	61%	31%
Cherqui et al, 2006	PC	3.33 ± 1	8 (30%)	93%	64%	93%	64%	NA	NA
Chen et al, 2008	RS	2.1	NA	91%	NA	NA	NA	61%	NA
Cai et al, 2008	RS	3.99	2 (6%)	95.4%	NA	NA	NA	56.2%	46%
Sarpel et al, 2009	PC	4.3	Not Clear	100%	90%	NA	NA	95%	50%
Yoon et al, 2010	PC	3.1	21 (30.4%)	90.4%	NA	90.4%	60%	NA	60%
Truant et al, 2011	RS	2.9 ± 1.2	16 (44.4%)	89%	NA	89%	46%	NA	46%
Akishige et al, 2013	RS	2 ± 08	NA	NA	NA	NA	NA	NA	NA
Memeo et al, 2014	PSM	3.2 (0.9–11)	25 (55%)	88%	80%	ND	ND	59%	19%
Ahn et al, 2014	PSM	2.6–1.5	12 (23.5%)	ND	ND	ND	ND	80.1%	67.8%
Komatsu et al, 2016	RC	4.7 (2.3–1.)	ND	ND	ND	73.4%	50.3%	ND	ND
Chen et al, 2017	PSM	ND	ND	100%	91.5%	92.6%	72.2%	ND	ND
Junhua et al, 2017	RC	3.9 (1.53)	ND	ND	ND	ND	ND	ND	ND
Hong et al, 2018	PSM	4.3 (1–10)	ND	100%	95.5%	86.7%	72.9%	ND	ND
Yusuke et al, 2018	RC	1.8 (0.4–4.)	ND	ND	ND	ND	ND	ND	ND
Rhu et al, 2018	PSM	3.1 (1.8)	6 (11.3%)	96.8%	77.8%	94.5%	68.3%	94.5%	62.5%
Yoon et al, 2019	PSM	2.83 (±1.28)	ND	98.1%	81.0%	87.0%,	62.0%	78.6%	49.1%
Peng et al, 2019	PSM	3 (3–5)	ND	95.8%	71.9%	77.0%	51.4%	ND	ND
Onoe et al, 2019	RC	1.8 (0.4–4.5)	ND	ND	ND	ND	ND	ND	ND
Yamamoto et al, 2019	PSM	1.7 (1–4.2)	ND	ND	ND	82.0%	58.9%	52.6%	24.0%
Goh et al, 2019	PSM	2 (1.1–2.8)	ND	100%	99.0%	93%	91.2%	89%	82%

DFS = disease-free survival, Mo = month, NA ***=*** not available, ND = no data, OS = overall survival, PSM = propensity-score matching, RC = retrospective comparative, RS ***=*** retrospective study.

The ranges of DFS rates at 1 year, 3 years and 5 years were 46% to 99%, 46% to 91.2%, and 19% to 82%, respectively (Table 4).

### 3.3 Intraoperative resection

We observed that most of the minor resections were segmentectomies 338/1363, partial-resections 262/1321 or lateral left sectionectomies 111/1363. Resections were preformed in most of the publications, there were only 4 studies where the data about minor resections were not reported. Most of the major resections were right and left hepatectomies, right hepatectomy 102/1363, left hepatectomy 109/1363 and trisectionectomy 50/1363, trisegmentectomy 61/1363. Only 5 studies did not have data on major resections (Table 5).

**Table 5:** Type of resections.^[14–36]^.

	Major resection				Minor resection		
Study (year)	Ri-hep	Le-hep	Tri sec	Tri-seg	P-Seg	Partial- hepatectomy	LL-sectionectomy
Shimada et al, 2001	0	0	0	0	0	10	7
Laurent et al, 2003]	0	0	0	3	7	3	0
Kaneko et al, 2005	0	0	0	0	0	20	10
Cherqui et al, 2006	1	0	0	0	5	17	3
Chen et al, 2008	4	8	0	7	97	0	0
Cai et al, 2008	0	3	0	8	0	17	3
Sarpel et al, 2009	0	0	0	0	0	20	0
Yoon et al, 2010	6	2	0	1	10	44	6
Truant et al, 2011	0	0	0	0	14	22	0
Akishig et al, 2013	3	3	0	5	22	23	0
Memeo et al, 2014	0	0	0	15	11	19	0
Ahn et al, 2014	0	2	0	0	32	3	14
Komatsu et al, 2016	8	0	0	0	14	16	0
Chen et al, 2017	16	9	6	3	31	0	16
Junhua et al, 2017	11	26	10	19	27	33	6
Hong et al, 2018	13	23	0	0	0	0	0
Yusuke et al, 2018	NA	NA	NA	NA	NA	NA	NA
Rhu et al, 2018	NA	NA	NA	NA	NA	NA	NA
Yoon et al, 2019	40	32	33	0	57	11	44
Peng et al, 2019	NA	NA	NA	NA	NA	NA	NA
Onoe et al, 2019	NA	NA	NA	NA	0	4	1
Yamamoto et al, 2019	NA	NA	NA	NA	NA	NA	NA
Goh et al, 2019	0	1	1	0	11	0	7

Hep = hepatectomy, Le = left, LL-Sectionectomy = lateral lobe sectionectomy, NA ***=*** not available, P-Seg = partial segmentectomy, Ri = right, TriSec = trisectionectomy, Tri-seg = trisegmentectomy.

## 4 Discussion

There have been several systemic reviews, meta-analysis and articles published in last years to investigate the role of laparoscopic liver hepatectomy.^[36,38,39,42,43]^ The development of laparoscopic surgery in the past 3 decades has had a major impact on clinical practice. Laparoscopic hepatectomy became possible with new advancement in surgical skills and technology that allow easy and safe in intra-operative and bleeding control. The advantages of laparoscopic over open hepatectomy include fast recovery, shorter hospital stay, less postoperative complications, decreased infections and better cosmetic outcomes.^[40,41]^ The progressive spread of laparoscopic liver resection and the developments of new dedicated technologies have led to the need for redefining the role of laparoscopy in the field of liver resection. In this review we focused on major outcomes of LLR for HCC in order to illustrate the feasibility and advantages of laparoscopic liver surgery. There was no difference in terms of surgical margin, overall survival or disease-free survival when the laparoscopic approach was used compared to the conventional open approach. The results showed that the laparoscopic approach has better short-term outcomes with less blood loss, and shorter hospital stays. However, this information must be interpreted with caution.

Among the selected publications, that include large series of matched patients Yoon et al reported on 217 patients^[32]^ for which LLR shows comparable results to OLR. Overall postoperative morbidity rates were significantly lower for the LLR group. The mortality rates are very low compared to OLR.

Overall morbidity does not exceed 25%, except for the study by Hong et al ,^[29]^ in which the morbidity was 30.6% in the LMR group. The mortality rates are 0% for LMR compared to OMR's 3.1%. Overall complications were lower with LMR than OMR.

Cirrhotic liver resection is challenging even for expert surgeons. The challenges include postoperative hemorrhage, which is associated with low platelet counts and portal hypertension. Careful selection of cirrhotic HCC patients for surgery is of vital importance. In the present study, all the included patients had well-preserved liver functions and had adequate volumes of FLR. According to Ribero and Wakabayashi an FLR of > 40% of TLV is the safe limit for liver resection for cirrhotic HCC patients.^[44,45]^ Another report (by Marco Vivarelli and colleagues) reported prophylaxis for venous thromboembolism (VTE) after hepatocellular carcinoma resection in Cirrhosis patients and their report showed that Prophylaxis is safe in cirrhotic patients and patients had low incidence of postoperative thromboembolic complications.^[9]^ Ramacciato and colleagues reported Hepatic resection treatment for cirrhotic patients the results with Short term were completely acceptable results with low mortality rate 5%, and long term results were high recurrence rate for patients with cirrhosis.^[51]^

According to Llovet and colleagues’ initial report, Number of cases with cirrhosis in Western area, the best candidates were described as those with a single tumor, bilirubin <1 mg/dl and without portal hypertension (defined by hepatic venous pressure gradient [HVPG] <10 mm Hg or platelet count >100,000/μL). In this case, overall survival following LR was close to that observed after LT (5-year OS of 74% for LR and 69% for LT).^[47,48]^

The extent of surgery and the volume of the future liver remnant can be estimated by calculating the volumes of the removed part, as well as the remnant part as a fraction of the total liver volume. These volumes are calculated using routine CT and MRI with semi-automatic software. It has been demonstrated that a remnant liver volume of approximately 25% to 30% of the total liver volume in patients without cirrhosis, and 40% in case of cirrhosis, is required before a major hepatectomy to minimise the risk of postoperative liver failure.^[49]^ Addition, Liver function is usually estimated by the Child-Pugh score and patients with Child-Pugh B or C are at a high risk of liver failure even after a minor hepatectomy. Recently, a preoperative model for end-stage liver disease (MELD) scores higher than 9 was associated with low OS after LR, leading to the incorporation of the MELD score into the EASL guidelines for treatment allocation.^[50]^

Komatsu et al^[26]^ reported more Clavien I–II complications than Clavien III-V complications. Intra-operative outcomes Rhu et al^[31]^ reported a significantly higher total operation time, in fact it had the longest operation time, and blood loss was not clear. Onoe et al^[34]^ reported the longest duration of hospitalization with a median of 10 days.

LLR also reduces adhesions due to previous liver resections, that may account for the remarkable increase of difficulty in these types of liver transplants (LT). A study published in 2009 by Laurent et al^[46]^ comparing intra-operative LT after ORL and LLR, showed that the latter was associated with reduced blood loss, a reduced need for transfusions and a shorter duration of hepatectomy.

The previous study described how robotic surgery has demonstrated a similar blood loss and complication rate compared with the laparoscopic procedure. Robotic liver resection is emerging as a valid alternative to the laparoscopic approach. One study published by Chen et al.^[27]^ reviewed the largest series of RLR for HCC, none of which found any difference in overall survival and DFS between RLR and ORL or between RLR and OLR.

The study conducted by Dagher et al^[37]^ included an analysis of results separating the first 25 resections into “early experience” and “recent experience,” the results showed a significant improvement of surgical and postoperative results in the “recent experience” group. Finally, Major LLR may be associated with a relatively high risk of excessive intra-operative bleeding, conversion and liver failure, especially for patients with a history of hepatectomy.

## 5 Conclusion

In the last decades, surgical techniques in both peroperative and postoperative management have improved, as well as patient selection. Laparoscopic hepatectomy has proven to be a safe and feasible treatment option for HCC and LH is now considered standard procedure for HCC in many centers globally. With the advancement of surgical techniques, laparoscopic liver resection is being performed much more recently even for tumors in difficult locations. LH outcomes were comparable to other procedures and achieved acceptable short-long-term survival outcomes. Better patient selection and experienced laparoscopic surgeons are the main points for success and favorable surgical outcomes. Laparoscopic techniques are associated with improved rates of surgical site infections, postoperative complications, and shorter hospital stays, without compromising oncological outcomes for cancer resections.

Hopefully these studies will encourage a responsible approach for these complex and difficult patients, though it is interesting to note that while laparoscopic liver resection is still in its infancy, it may be the best technique in the future.

## Author contributions

**Conceptualization:** Xiujun Cai.

**Data curation:** Maher Hendi, Jiemin Lv, Xiujun Cai.

**Formal analysis:** Xiujun Cai.

**Investigation:** Maher Hendi.

**Methodology:** Maher Hendi, Xiujun Cai.

**Project administration:** Maher Hendi, Xiujun Cai.

**Software:** Maher Hendi.

**Supervision:** Xiujun Cai.

**Validation:** Maher Hendi.

**Visualization:** Maher Hendi.

**Writing – original draft:** Maher Hendi, Xiujun Cai.

**Writing – review & editing:** Maher Hendi, Jiemin Lv, Xiujun Cai.
